# The anti-aging effect of vitamin D and vitamin D receptor in *Drosophila* midgut

**DOI:** 10.18632/aging.205518

**Published:** 2024-02-07

**Authors:** Joung-Sun Park, Hyun-Jin Na, Yung-Jin Kim

**Affiliations:** 1 Institute of Nanobio Convergence, Pusan National University, Busan 46241, Republic of Korea; 2 Department of Molecular Biology, Pusan National University, Busan 46241, Republic of Korea; 3 Aging and Metabolism Research Group, Korea Food Research Institute, Wanju 55365, Republic of Korea

**Keywords:** *Drosophila*, vitamin D, vitamin D receptor, anti-aging, intestinal stem cell

## Abstract

Adult stem cells are pivotal for maintaining tissue homeostasis, and their functional decline is linked to aging and its associated diseases, influenced by the niche cells’ environment. Age- and cancer-related reduction of vitamin D and its receptor levels are well documented in human clinical studies. However, the mechanisms through which the vitamin D/vitamin D receptor pathway contributes to anti-aging and extends life expectancy are not well understood. In this study, we aimed to determine the protective role of the vitamin D/vitamin D receptor pathway in differentiated enterocytes (ECs) during intestinal stem cell (ISC) aging. By utilizing a well- established *Drosophila* midgut model for stem cell aging biology, we revealed that vitamin D receptor knockdown in ECs induced ISC proliferation, EC death, ISC aging, and enteroendocrine cell differentiation. Additionally, age- and oxidative stress-induced increases in ISC proliferation and centrosome amplification were reduced by vitamin D treatment. Our findings suggest a direct evidence of the anti-aging role of the vitamin D/vitamin D receptor pathway and provides insights into the molecular mechanisms underlying healthy aging in *Drosophila*.

## INTRODUCTION

Adult stem cells are fundamental to maintaining organizational homeostasis [1]. The gradual decline in the function of adult stem cells is closely associated not only with tissue and organismal aging but also with age-related diseases such as diabetes and cancer [1, 2]. Adult stem cells reside within the cellular microenvironment, surrounded by heterogeneous cell populations [1, 2]. It is well documented the importance of this microenvironment for maintaining the functional integrity of adult stem cell [1, 2]. Investigating the properties of the niches that promote the aging of tissue-resident stem cells could offer a new perspective on tissue homeostasis, organismal aging regulation, and age-related disease prevention.

*Drosophila*, known for its short lifespan and a genetically modifiable midgut functionality, is a well-established organism for aging studies, including research on adult stem cells, their niches, and aging-related changes [3–5]. In the adult *Drosophila* midgut, intestinal stem cells (ISCs, Delta-positive cell) are the only mitotic cells that generate two differentiated progeny cell types: absorptive enterocytes (ECs) via stronger Notch (N) signal enteroblasts (EBs) and secretory enteroendocrine (EE) cells via a weak N signal EBs [4, 5]. These four types of cells are distinguished by the expression of cell-specific markers, including Delta (ISCs), PH3 (dividing ISC), esg- green fluorescent protein (GFP; ISCs and EBs), Su-GFP (EBs), Pdm and Myo-GFP (ECs), and Prospero (Pros) and Pros-GFP (EEs) [3–7].

The effects of aging, high metabolism, and infection induce cellular intrinsic and extrinsic oxidative stresses, accelerating the proliferation of ISCs [8–13]. ISC hyperproliferation is linked to increased DNA damage and supernumerary centrosomes, characteristic hallmarks of cancer in aged guts and those exposed to oxidative stress [14–16]. The internal pathways of ISCs (e.g., N, EGFR, PVR, ATM/ATR), paracrine factors (e.g., Wg, Upds), and visceral muscles regulate ISC proliferation [5–10, 17–33].

Age-related reductions in the vitamin D (VitD) synthesis and VitD receptor (VDR) expression are linked to age-related diseases such as cancer [34–36]. However, only a few studies on the role of VitD/VDR and their exact mechanism of action in adult stem cells exist, warranting further research. VitD acts by binding to VDR and the VitD response element (VDRE), which is located on several VitD target genes [37]. In *Drosophila*, *Hr96* is a transcription factor orthologous to human VDR [38]. During mid-embryogenesis stages, *Hr96* is mainly expressed in the excretory organs, fat body, and central nervous system [39] and is primarily stimulated by the ecdysone hormone, a primary factor in mediating molting and metamorphosis [38]. *Hr96* is activated by small lipophilic compounds produced from dietary signals and metabolic intermediates and regulates developmental processes and cellular metabolisms [40]. *Hr96* recognizes xenobiotic substances and triggers the expression of detoxification- and clearance-related genes [41]. Furthermore, *Hr96 plays* an essential role in lipid metabolism by detecting triacylglycerol levels, facilitating their degradation, and regulating the catabolism of cholesterol through the regulation of genes associated with cholesterol uptake, storage, and intracellular trafficking [42, 43]. A recent study reported that Hr96 is involved in new intestinal cell differentiation by altering the level and duration of N signaling in the adult *Drosophila* intestine [44]. However, the role of *Drosophila* Hr96 in maintaining homeostasis in the adult intestine remains unclear.

This study aimed to determine the protective role of VitD/VDR in differentiated ECs during ISC aging using the adult *Drosophila* intestine model.

## RESULTS

### VDR knockdown in ECs induces ISC proliferation

**Figure 1:**
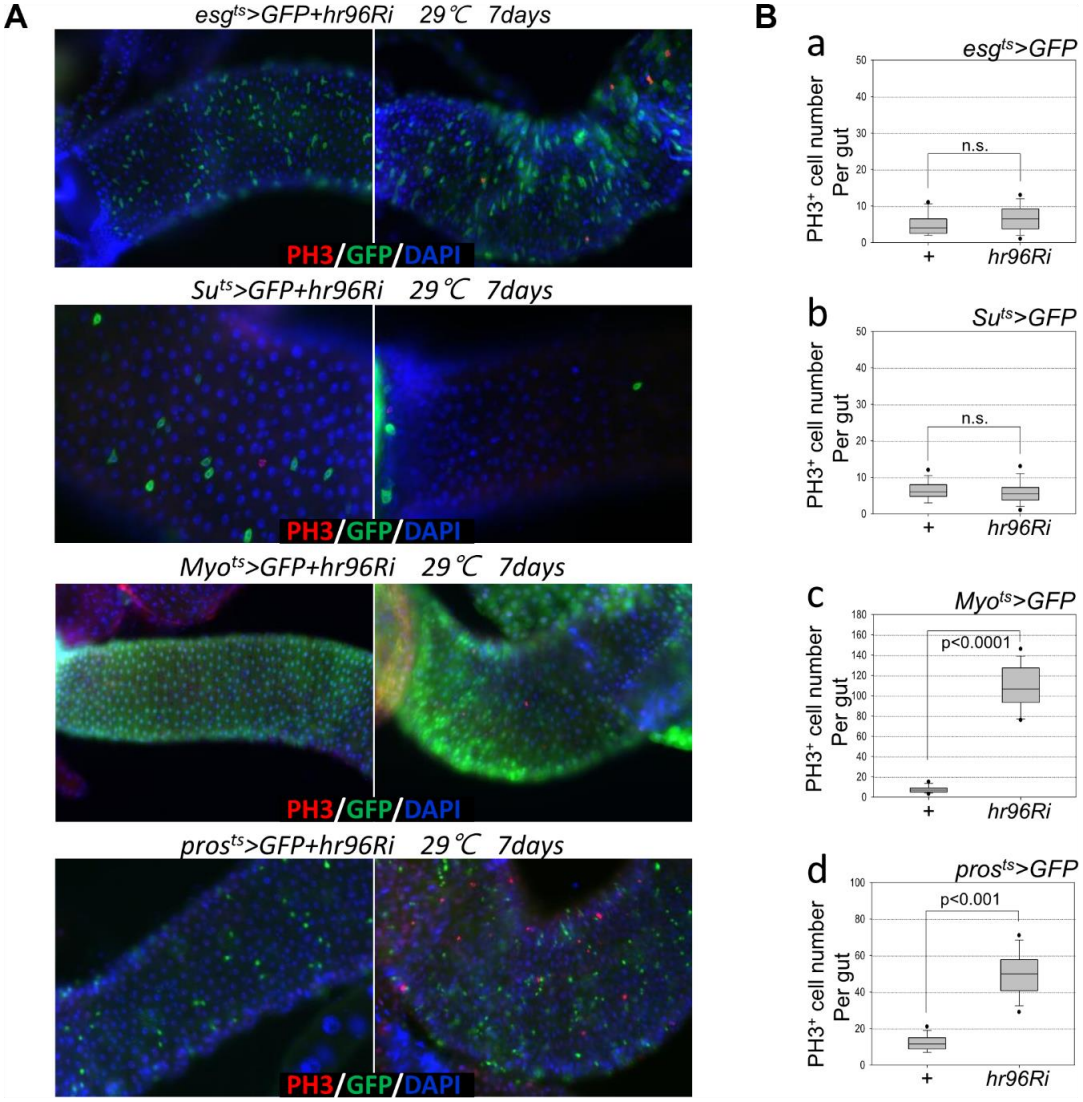
**Effects of intestinal cell type-specific VDR knockdown on ISC proliferation.** (**A**, **B**) ISC and EB (*esg^ts^*), EB (*Su^ts^*), EC (*Myo^ts^*), and EE (*pros^ts^*)-specific knockdown of VDR induces ISC division. Flies carrying *esg^ts^>GFP* and *esg^ts^>GFP+VDRRi*, *Su^ts^>GFP* and *Su^ts^>GFP*+*VDRRi*, *Myo^ts^>GFP* and *Myo^ts^>GFP+VDRRi*, or *pros^ts^>GFP* and *pros^ts^>GFP*+*VDRRi* genotypes were cultured for 4 days at 29° C. The midguts of the flies were dissected, fixed, and labeled with anti-PH3 (red) and anti-GFP (green) antibodies and DAPI (blue). The original magnification is 200×. (**B**) The number of PH3^+^ cells in the midgut with ISC and EB- (*esg^ts^*), EB- (*Su^ts^*), EC- (*Myo^ts^*), and EE (*pros^ts^*)-specific VDR knockdown. Gut samples of *esg^ts^>GFP* and *esg^ts^>GFP+VDRRi* (**a**), *Su^ts^>GFP* and *Su^ts^>GFP*+*VDRRi* (**b**), *Myo^ts^>GFP* and *Myo^ts^>GFP+VDRRi* (**c**), or *pros^ts^>GFP* and *pros^ts^>GFP*+*VDRRi* (**d**) flies maintained at 29° C for a week were labeled with anti-PH3 (red) and anti-GFP (green) antibodies and DAPI (blue). PH3^+^ cell numbers were determined in the entire gut under a microscope. *P*-values were determined using Student’s *t*-test. *p* < 0.001, *p* < 0.0001. n.s., no significant differences.

To investigate the role of VDR in the adult *Drosophila* intestine, flies with ISC/EB-, EB-, EC-, or EE-specific VDR knockdown were generated using flies with the *esg^ts^*>*GFP*, *Su^ts^*>*GFP*, *Myo^ts^*>*GFP*, or *pros^ts^*>*GFP* genotypes. esg-GFP and PH3 signals were examined, and the number of esg-GFP^+^ and PH3^+^ cells in the guts of *esg^ts^*>*GFP* and *esg^ts^*>*GFP+VDRRi* flies kept at 29° C for 7 days did not significantly differ (Figure 1A, 1^st^ panel and 1B a). In addition, Su-GFP and PH3 signals were examined, and the number of PH3^+^ cells in the guts of *Su^ts^*>*GFP* and *Su^ts^*>*GFP+VDRRi* flies kept at 29° C for 7 days did not significantly differ (Figure 1B b); however, the number of Su-GFP^+^ cells significantly decreased (Figure 1A, 2^nd^ panel). In flies with the *Myo^ts^*>*GFP* or *pros^ts^*>*GFP* genotypes, a dramatic increase in the number of PH3+ cells was detected (Figure 1A 3^rd^ and 4^th^ panel, and 1B c and d). Quantification of the number of PH3^+^ cells per gut revealed values of 6.58 in the *esg^ts^>GFP+VDRRi* flies (*N* =14, *n* = 92; *N* indicates the number of guts, and *n* indicates the number of PH3^+^ cells), 4.85 in the *esg^ts^>GFP* flies (*N* = 13, *n* = 36) (Figure 1B a), 5.7 in the *Su^ts^>GFP+VDRRi* flies (*N* =14, *n* = 80), 6.36 in the *Su^ts^>GFP* flies (*N* = 14, *n* = 89) (Figure 1B b), 108.57 in the *Myo^ts^>GFP+VDRRi* flies (*N* =14, *n* = 1520), 7.43 in the *Myo^ts^>GFP* flies (N = 14, n = 104) (Figure 1B c), 49.86 in the *pros^ts^>GFP+VDRRi* flies (*N* =14, *n* = 698), and 12 in the *pros^ts^>GFP* flies (*N* = 14, *n* = 168) (Figure 1B d). These results indicate that the EC-specific knockdown of VDR markedly increases ISC proliferation.

### VDR knockdown in ECs induces EC death

**Figure 2:**
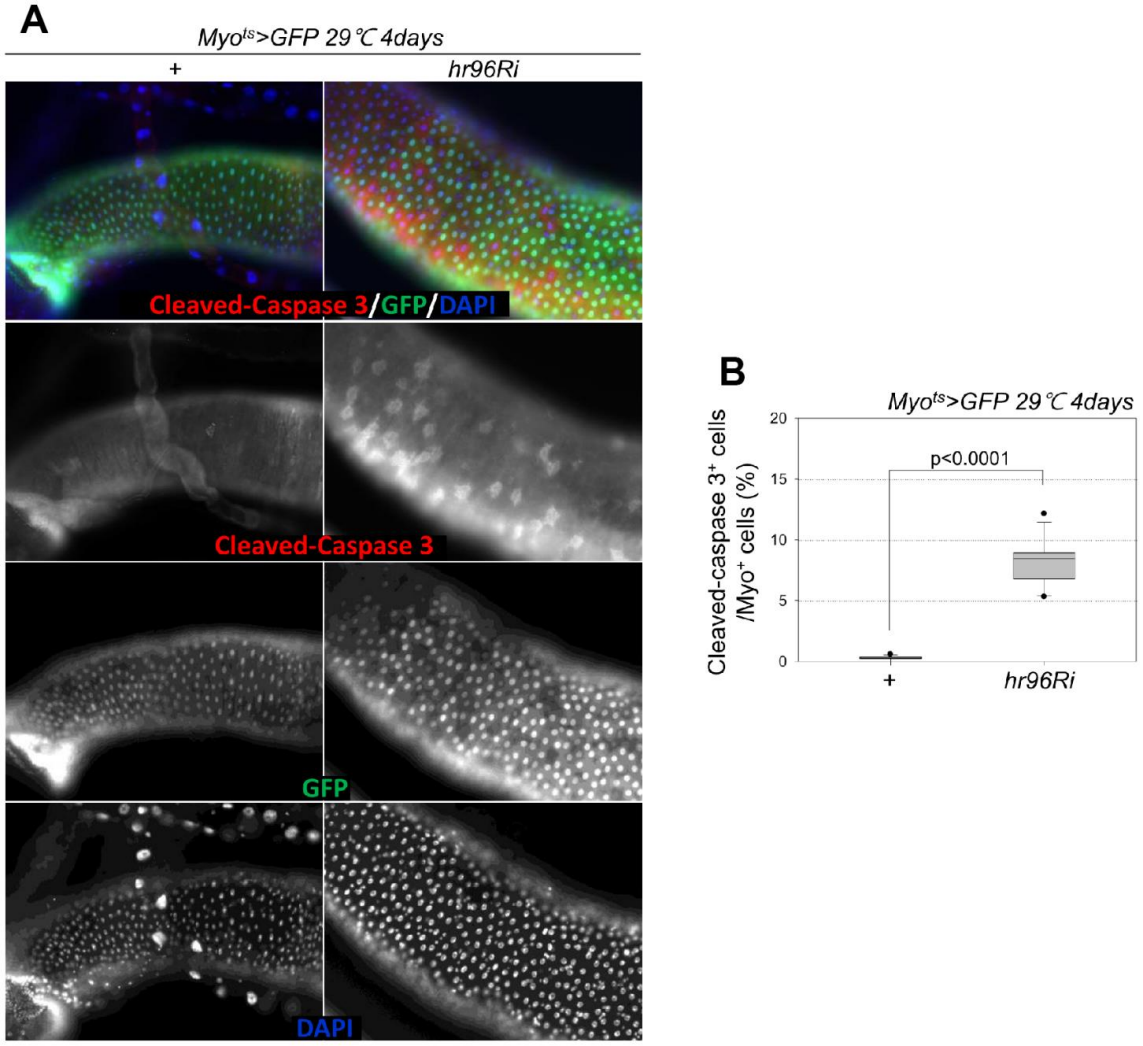
**Effect of EC-specific VDR knockdown on EC cell death.** (**A**) EC-specific knockdown of VDR induces cleaved caspase-3 in ECs. Flies carrying the *Myo^ts^>GFP* or *Myo^ts^>GFP*+*VDRRi* genotypes were cultured at 29° C for 4 days. The guts of flies were dissected and labeled with anti-GFP (green) and anti-cleaved caspase-3 (red) antibodies and DAPI (blue). Original magnification is 400×. (**B**) Frequency of cleaved-caspase 3^+^ cells per Myo^+^ (strong and weak GFP^+^) large cell (ECs). Three-day-old females were shifted to 29° C for 4 days, and dissected guts were immunostained with anti-GFP (green) and anti-cleaved caspase 3 (red) antibodies and DAPI (blue). Cleaved caspase 3^+^ cell numbers were determined in the Myo^+^ cells of these guts. Data (mean ± standard error) in the *Myo^ts^>GFP* or *Myo^ts^>GFP*+*VDRRi* flies were collated from 4716 and 5914 Myo^+^ cells of 15 guts each. *P*-values were determined using Student’s *t*-test. *p*<0.0001 compared with *Myo^ts^>GFP* flies.

To assess the role of VDR in EC death, cleaved caspase-3 signals were examined; they were increased in the Myo-GFP^+^ cells of the guts of *Myo^ts^*>*GFP* and *Myo^ts^*>*GFP+VDRRi* flies kept at 29° C for 4 days. Very weak cleaved caspase-3 signals were detected in the ECs of *Myo^ts^*>*GFP* flies (Figure 2A). In contrast to the signal in wild-type *Myo^ts^*>*GFP* flies, the cleaved caspase-3 signal was markedly increased in the Myo^+^ cells (ECs) of *Myo^ts^*>*GFP+VDRRi* flies (Figure 2A). Subsequently, the ratio of cleaved caspase-3^+^ in Myo-GFP^+^ cells was quantified. A significant increase in EC death was observed in the gut following EC-specific VDR knockdown (Figure 1B). These results indicate that VDR is required for EC survival under normal conditions.

### VDR knockdown in ECs induces ISC aging

**Figure 3:**
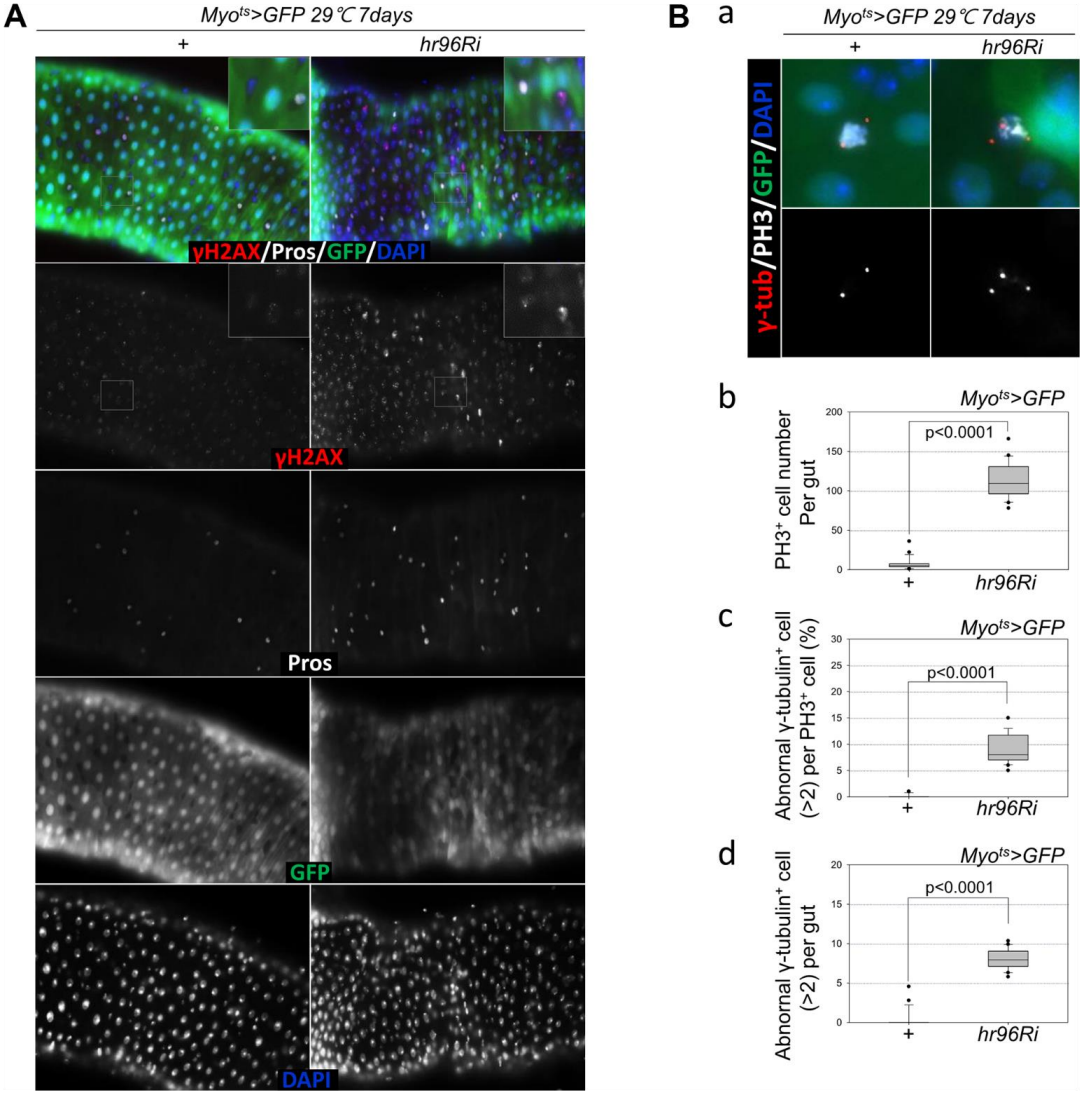
**EC-specific VDR knockdown increases age-related ISC phenotypes.** (**A**) EC-specific VDR knockdown induces DNA damage accumulation in ISCs and their progenitors. Flies carrying the *Myo^ts^>GFP* or *Myo^ts^>GFP*+*VDRRi* genotype were incubated at 29° C for 7 days. The guts of flies were dissected and labeled with anti-γH2AvD (red), anti-Pros (white), and anti-GFP (green) antibodies and DAPI (blue). (**B**) EC-specific VDR knockdown causes centrosome amplification in mitotic ISCs. Flies carrying the *Myo^ts^>GFP* or *Myo^ts^>GFP*+*VDRRi* genotype were incubated at 29° C for 7 days. (**a**) The entire guts of the flies were dissected, fixed, and labeled with anti-γ-tubulin (red), anti-PH3 (white), and anti-GFP (green) antibodies and DAPI (blue). The original magnification is 400×. (**b**–**d**) The number of mitotic ISCs with supernumerary centrosomes (>2) in the midguts of *Myo^ts^>GFP* or *Myo^ts^>GFP*+*VDRRi* flies increased. (**b**) EC-specific VDR knockdown increases mitotic ISCs in the guts. (**c**) Frequency of cells with supernumerary centrosomes per mitotic ISC. (**d**) Number of cells with supernumerary centrosomes per midgut. Three-day-old female flies were cultured at 29° C for 7 days, and then their dissected guts were fixed and immunostained with anti-γ-tubulin (red), anti-PH3 (white), and anti-GFP (green) antibodies and DAPI (blue). In these guts, the number of abnormal centrosomes in the PH3^+^ cells were determined. Data (mean ± SD) in *Myo^ts^>GFP* or *Myo^ts^>GFP*+*VDRRi* flies were collated from 154 and 2281 mitotic cells of 21 and 20 guts, respectively. *P*-values were calculated using Student’s *t*-test. *P* < 0.0001 compared with *Myo^ts^>GFP* flies.

To assess the implications of excessive ISC proliferation due to EC-specific VDR knockdown-induced EC death, damage accumulation in ISCs was analyzed using antibodies against γH2AvD (a molecular marker of DNA double strand breaks) [14, 45], and Pros (an EE marker). The γH2AvD signal was exceedingly low in the Myo-GFP^-^ and Pros^-^ cells (ISCs) of *Myo^ts^>GFP* flies (Figure 3A, left panel); however, γH2AvD foci were markedly increased in the Myo-GFP^-^ and Pros^-^ ISCs of *Myo^ts^>GFP+VDRRi* flies (Figure 3A, right panel). This indicates that the EC-specific knockdown of VDR could induce DNA damage accumulation in ISCs. Centrosome amplification (a hallmark of cancer cells) was also examined using anti-γ-tubulin and anti-PH3 antibodies. In control files, two centrosomes in mitotic ISCs (PH3^+^ cells) were detected; however, mitotic ISCs with 3–12 abnormal centrosomes were detected in the EC-specific VDR-knockdown flies carrying the *Myo^ts^>GFP+VDRRi* genotype (Figure 3B a). Subsequently, the number of PH3^+^ cells per gut was quantified, and 114.05 were observed in *Myo^ts^>GFP+VDRRi* flies (*N* = 20, *n* = 2281), while 7.33 were observed in *Myo^ts^>GFP* flies (*N* = 21, *n* = 154) (Figure 3B b). In addition, the frequencies of these mitotic ISCs with supernumerary centrosomes (>2) were quantified, revealing values of 8.03 and 0.35% in *Myo^ts^>GFP+VDRRi* and *Myo^ts^>GFP* flies, respectively (Figure 3B c). The number of mitotic ISCs with supernumerary centrosomes (>2) per gut was 9.25 in the *Myo^ts^>GFP+ VDRRi* flies and 0.09 in the *Myo^ts^>GFP* flies (Figure 3B d). These results show that VDR inhibition resulted in DNA damage accumulation and a higher incidence of centrosome amplification in ISCs.

### VDR knockdown in ECs induces EE differentiation

**Figure 4:**
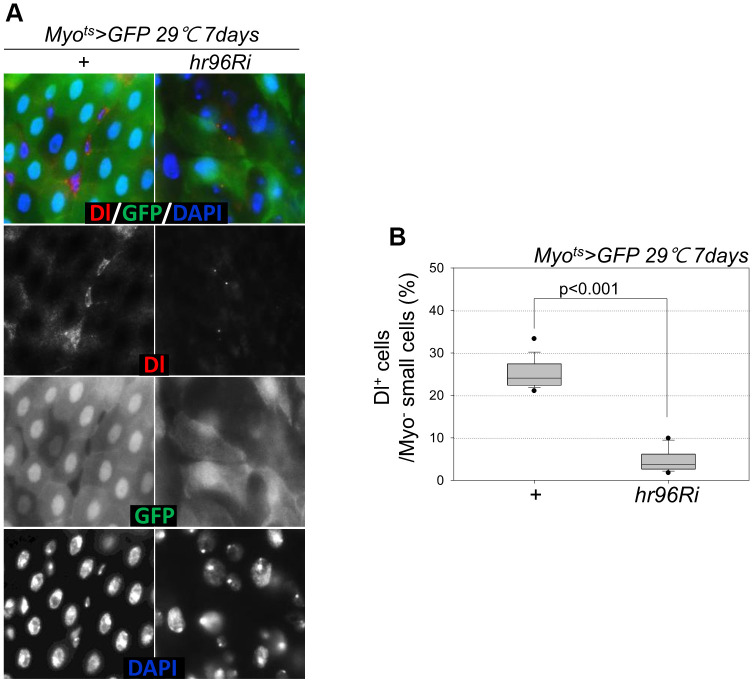
**EC-specific VDR knockdown decreases the number of Dl^+^ cells.** (**A**) EC-specific knockdown of VDR decreases the number of Delta^+^ cells. Flies carrying the *Myo^ts^>GFP* or *Myo^ts^>GFP*+*VDRRi* genotypes were incubated at 29° C for 1 week. The whole guts of flies were dissected, fixed, and labeled with anti-Dl (red) and anti-GFP (green) antibodies and DAPI (blue). The original magnification is 400×. (**B**) Frequency of Dl^+^ cells per Myo^-^ small cell. Three-day-old females were shifted to 29° C for 1 week, and their dissected guts were fixed and immunostained with anti-Dl (red) and anti-GFP (green) antibodies and DAPI (blue). The number of Dl^+^ cells in the Myo^-^ small cells was determined in these guts. Data (mean ± standard error) in *Myo^ts^>GFP* or *Myo^ts^>GFP*+*VDRRi* flies were collated from 882 and 1192 Myo^-^ cells of 16 and 15 guts, respectively. *P*-values were determined using Student’s *t*-test. *P* < 0.0001 compared with *Myo^ts^>GFP* flies.

**Figure 5:**
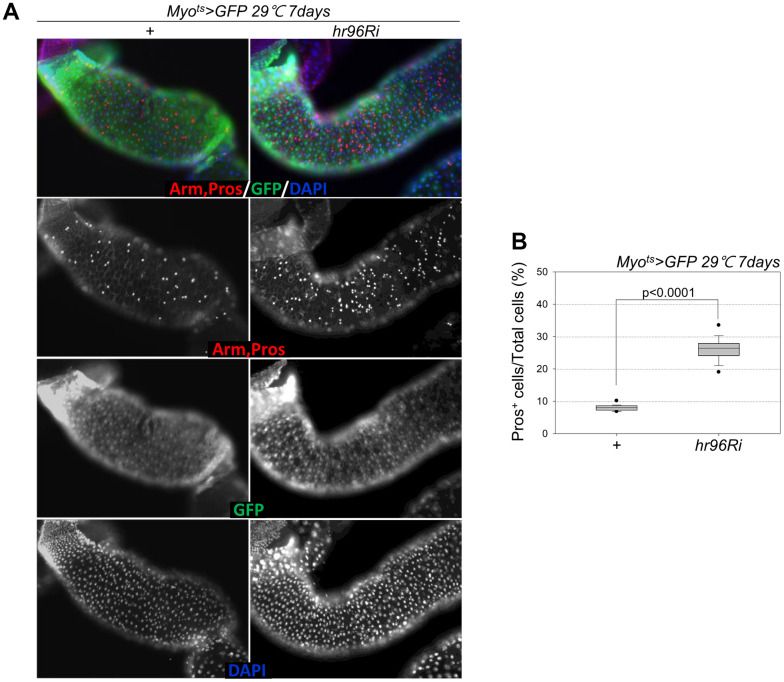
**EC-specific VDR knockdown increases the number of Pros^+^ cells.** (**A**) EC-specific VDR knockdown increases Pros^+^ cell number. Flies carrying the *Myo^ts^>GFP* or *Myo^ts^>GFP*+*VDRRi* genotypes were incubated at 29° C for 1 week. The whole guts of flies were dissected, fixed, and labeled with anti-Pros (red) and anti-GFP (green) antibodies and DAPI (blue). The original magnification is 200×. (**B**) Frequency of Pros^+^ cells per total cells. Three-day-old females were shifted to 29° C for 1 week, and their dissected guts were fixed and immunostained with anti-Pros (red) and anti-GFP (green) antibodies and DAPI (blue). The Pros^+^ cell numbers were recorded with respect to the total cells of these guts. Data (mean ± standard error) in *Myo^ts^>GFP* or *Myo^ts^>GFP*+*VDRRi* flies were collated from 7459 and 7992 total cells of 19 guts each, respectively. *P*-values were determined using Student’s *t*-test. *P*<0.0001 compared with *Myo^ts^>GFP* flies.

Following EC-specific VDR knockdown, the increase in Dl^+^ cell numbers in the gut was analyzed using an anti-Dl antibody (Dl is a molecular marker of ISCs). Unlike the previously reported increase in the number of strong Dl^+^ cells (ISC-EB-EC) following the age-related increase in ISC proliferation [8–10], very weak Dl^+^ cells and a marked decrease in Dl^+^ cell numbers in guts with EC-specific VDR knockdown were observed (Figure 4A, 4B). Therefore, to evaluate ISC differentiation in guts with EC-specific VDR knockdown, the number of Pros^+^ cells in these guts was analyzed using an anti-Pros antibody since Pros is a molecular marker of EE. The number of EEs significantly increased in the guts of *Myo^ts^*>*GFP+VDRRi* flies compared with the guts of wild-type *Myo^ts^*>*GFP* flies (Figure 5A and Supplementary Figure 1). Furthermore, the EE ratio in the midgut was quantified. Significant increases in EEs were detected in the guts of EC-specific VDR knockdown flies (Figure 5B and Supplementary Figure 1). This indicates that VDR knockdown in ECs induces an excessive increase in EE numbers. Additionally, we evaluated whether VDR knockdown-induced EE differentiation was ameliorated by VitD treatment. First, to this end, it was determined that VitD could activate the VDR pathway by feeding adult flies with VitD using an anti-hVDR antibody. That is, 7-day-old wild-type flies (*Oregon-R*) were fed with 100 nM 1α,25-Dihydroxyvitamin D_3_ for 24 h. In wild-type flies not fed 1α,25-Dihydroxyvitamin D_3_, VDRs were scattered throughout the intestinal cells, including ECs and EE cells (Supplementary Figure 2, left panels). In contrast, VDRs migrated to the nuclear membrane in the intestinal cells of flies fed 1α,25-Dihydroxyvitamin D_3_ (Supplementary Figure 2, right panels). Second, we determined whether VitD could activate the VDR pathway in *Myo^ts^>GFP* and *Myo^ts^>GFP+hr96Ri* flies (Supplementary Figure 3). As hypothesized, EC-specific VDR knockdown-induced EE differentiation was ameliorated by VitD treatment (Supplementary Figure 4). These results indicated that the VitD/VDR (Hr96) pathway in fly intestinal models functions similarly to that in mammals. Therefore, VDR is involved in EE differentiation under normal conditions.

### Inhibitory effect of VitD on age- and oxidative stress-related ISC proliferation and centrosome amplification in the midgut

**Figure 6:**
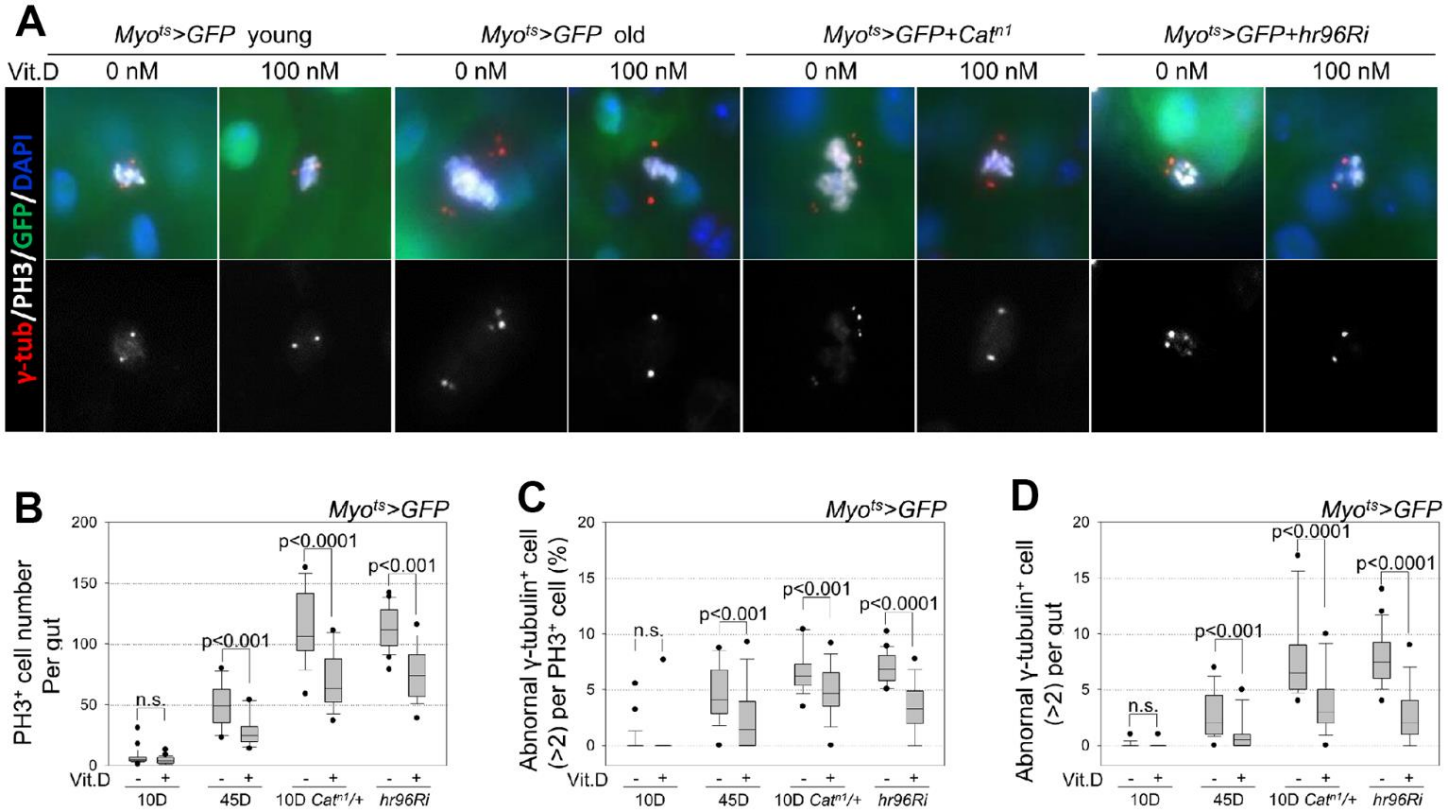
**Inhibitory effect of VitD on age- and oxidative stress-related accumulation of supernumerary centrosomes in midgut ISCs.** (**A**) The guts from 10-day-old *Myo^ts^>GFP* flies, 45-day-old *Myo^ts^>GFP* flies, 10-day-old *Myo^ts^>GFP*+*Cat^n1^* mutant flies, and 10-day-old *Myo^ts^>GFP+hr96Ri* flies, without or with 100 nM VitD feeding for 1 week were stained with anti-PH3 (white), anti-γ-tubulin (red), anti-GFP (green), and DAPI (blue). The original magnification is 400×. (**B**) The number of PH3^+^ cells in whole guts from 10-day-old *Myo^ts^>GFP*, 45-day-old *Myo^ts^>GFP*, 10-day-old *Myo^ts^>GFP*+*Cat^n1^*, and *Myo^ts^>GFP+hr96Ri* flies, with or without VitD feeding for 7 days was determined. Data (mean ± standard error) in 10-day-old *Myo^ts^>GFP*, 45-day-old *Myo^ts^>GFP*, 10-day-old *Myo^ts^>GFP*+*Cat^n1^*, and *Myo^ts^>GFP+hr96Ri* flies without VitD feeding were collated from 165, 823, 1835, and 2484 mitotic cells of 25, 17, 16, and 22 guts, respectively. Data (mean ± standard error) in 10-day-old *Myo^ts^>GFP*, 45-day-old *Myo^ts^>GFP*, 10-day-old *Myo^ts^>GFP*+*Cat^n1^*, and *Myo^ts^>GFP+hr96Ri* flies with VitD feeding were collated from 126, 489, 1235, and 1445 mitotic cells of 28, 18, 18, and 19 guts, respectively. n.s., no significant difference from the control (*p* > 0.05). (**C**) The frequency of supernumerary centrosomes (>2) per mitotic ISC in 10-day-old *Myo^ts^>GFP*, 45-day-old *Myo^ts^>GFP*, 10-day-old *Myo^ts^>GFP*+*Cat^n1^*, and *Myo^ts^>GFP+hr96Ri* flies with or without VitD feeding for 7 days. The centrosome numbers in mitotic ISCs (PH3^+^ and GFP^-^ cells) in the midgut were determined. n.s., no significant difference from the control (*p*>0.05). (**D**) The frequency of mitotic ISCs with supernumerary centrosomes per gut in 10-day-old *Myo^ts^>GFP*, 45-day-old *Myo^ts^>GFP*, 10-day-old *Myo^ts^>GFP*+*Cat^n1^*, and 10-day-old *Myo^ts^>GFP+hr96Ri* flies with or without VitD feeding for 1 week. Error bars represent standard error. *P*-values were calculated using Student’s t-test. n.s., no significant difference from the control (*p*>0.05).

The effect of VitD on age-related phenotypes (increased cell proliferation and centrosome amplification) in ISCs was investigated. To investigate the anti-aging effect of VitD, ISCs with or without 100 nM 1α,25-Dihydroxyvitamin D_3_ treatment were stained with antibodies against γ-tubulin (a centrosome marker), PH3 (a mitotic ISCs marker), and GFP (an ECs marker), and the number of cells displaying centrosome amplification was assessed. Supernumerary centrosomes were observed in 4.64% of mitotic ISCs in 45-day-old *Myo^ts^>GFP* flies (Figure 6A, 6C) and in 6.7% of 10-day-old *Myo^ts^>GFP+Cat^n1^* flies (Figure 6A, 6C), a model of intrinsic oxidative stress [8], compared with 0.35% in 10-day-old *Myo^ts^>GFP* flies (Figure 6A, 6C). The number of mitotic ISCs with supernumerary centrosomes per gut was 2.59 in 45-day-old *Myo^ts^>GFP* flies and 7.81 in 10-day-old *Myo^ts^>GFP+Cat^n1^* flies compared with 0.08 in 10-day-old *Myo^ts^>GFP* flies (Figure 6D). The age- and oxidative stress-related increase in the number of PH3^+^ cells was reduced by VitD treatment (Figure 6B). In addition, VitD treatment reduced the age- and oxidative stress-related increase in supernumerary centrosomes in 2.45 and 4.86% of mitotic ISCs in 45-day-old *Myo^ts^>GFP* and 10-day-old *Myo^ts^>GFP+Cat^n1^* flies, respectively (Figure 6A, 6C). The number of mitotic ISCs with supernumerary centrosomes per gut was reduced by 0.94 in 45-day-old *Myo^ts^>GFP* flies, and by 3.67 in 10-day-old *Myo^ts^>GFP+Cat^n1^* flies, whereas no change was observed in 10-day-old *Myo^ts^>GFP* flies (Figure 6A, 6C). Additionally, to determine whether the inhibitory effect of VitD on age-related phenotypes is associated with the VDR pathway, the VDR in ECs was knocked down. For EC-specific expression, 3-day-old *Myo^ts^>GFP* and *Myo^ts^>GFP+hr96Ri* flies were cultured at 29° C for 7 days. Compared with the untreated group, VitD treatment reduced mitotic ISCs and the number of mitotic ISCs with supernumerary centrosomes in the guts of *Myo^ts^>GFP* flies in which VDR was knocked down (Figure 6A, 6B). In contrast, VitD treatment did not affect the intestinal morphology of 10-day-old *Myo^ts^>GFP* flies. These results indicate that VitD can reduce age- and oxidative stress-induced centrosome amplification in adult ISCs *in vivo*.

## DISCUSSION

Aging and various cancers are associated with decreased VDR expression levels or increased polymorphisms. However, the exact role of VDR in each adult intestine cell type has not been identified. Our study demonstrated that the VitD/VDR pathway is required for intestinal homeostasis during normal differentiation and aging.

EC-specific VDR knockdown activates cleaved caspase-3 (an apoptotic signal), demonstrating that, under normal conditions, VDR is essential for the survival of differentiated ECs. ECs are continuously exposed to oxidative stress from external factors, including various foods and medicines [46]. In mammals, the VitD/VDR/Klotho/Nrf2 regulatory network can control redox homeostasis through antioxidants regulation [36]. In *Drosophila*, some EC protective factors have been established. First, the DNA damage response-related factors (MRN, ATM/ATR, and Chk1/Chk2) are major contributors in EC survival and the maintenance of intestinal homeostasis under the age-related increase of oxidative stress [47]. The antioxidant activity of VitD has been reported to reduce DNA replication stress severity, which is exerted through reduced constitutive γH2AX and ATM-S1981P expression levels [48]. Future research on the relationship between these two factors in the adult intestine is required. Second, *Su(var)3-9* and HP1a, which contribute to maintaining heterochromatin stability, protect ECs [45]. In addition, the non-stop identity complex, histones H3 and H4, and vitamin B6 protect differentiated ECs and promote survival [49–51]. A recent report suggested that the epigenomic effects of VitD are mediated by epigenetic factors such as IncRNAs, miRNAs, methylation, acetylation, and the interactions between VDR and certain genes such as SLC30A10 [52]. In the present study, HP1 was not detected in some ECs in EC-specific VDR knocked-down guts (Supplementary Figure 5). Further in-depth studies are needed to verify the role of VitD as an epigenetic regulator in ISCs and ECs.

Unlike a decrease of EE number in low level of VDR (Hr96 null mutant, *act>Hr96Ri*, *Pros^ts^>GFP*+*Hr96Ri*), ISC- and EB-specific ones (*esg^ts^>GFP*+*Hr96Ri*), on the other hand, EB-specific VDR knockdown caused a decrease in EB number (Supplementary Figure 6). In contrast, EC-specific VDR knockdown resulted in an increase in EE number and low levels of Delta^+^ and Myo^-^ cells, indicating that VDR is required for EC differentiation under normal conditions. EBs differentiate into ECs when the Dl level of ISCs is high [5]. In contrast, EBs differentiate into EEs when the Dl level of ISCs is low [5]. In addition, when dying, ECs release Upds, which bind to ISC receptors as ligands, increasing the division of ISCs, which in turn promotes differentiation into ISCs (high DL)-EBs (high Su)-ECs [20, 27, 53]. Contrary to previously reported studies, the present study found that EC-specific VDR knockdown induces EC death, promotes increased cell proliferation, and promotes differentiation into ISCs (low Dl)-EB (low Su)-EE. In addition, Pros^+^ and PH3^+^ cells (Prospero-positive ISCs) were identified in the EC-specific VDR knocked down gut (Supplementary Figure 7). Biteau and Jasper reported that prior to cell division, the expression of Pros in ISCs promotes EEs commitment [54]. Similarly, the data from the present study demonstrates that VDR expression in ECs is required for the optimal maintenance of an EC and EE lineage equilibrium. However, further research is required to understand the detailed mechanisms.

Furthermore, the present study also showed that EC-specific VDR knockdown increased ISC proliferation and DNA damage accumulation in ISCs/EBs, led to supernumerary centrosome accumulation, increased the numbers of EEs, and detected Pros^+^ and PH3^+^ cells. This demonstrates that low VDR levels in ECs promote the development of endocrine tumors (gastrointestinal carcinoid tumors) in the midgut, consistent with findings of previous studies. Case studies in patients with intestinal carcinoid tumors have shown that VitD deficiency is common [55–57]. An association between VDR polymorphisms and the risks and outcomes of several malignancies such as colon cancer has been reported [34, 35]. The levels of *VDR* mRNA expression in colorectal adenoma and adenocarcinoma tissues are lower than those in normal tissues [58]. Therefore, low intestinal VDR expression levels, and low plasma VitD levels in normal individuals or patients with intestinal diseases promote neoplasm development. This pathway could persist during the progression of adenoma to adenocarcinoma, carcinoid tumors, and colorectal cancer. Clarification of the potential association between the VitD/VDR pathway and the risk of intestinal cancer (carcinoid tumor) is therefore crucial.

Previously, we identified two safe anti-aging agents, metformin and β-hydroxybutyrate using the *Drosophila* midgut model [15, 24, 59]. However, further research on the development of safe anti-aging agents was required after that study. Therefore, here, for the first time, VDR was detected in the *Drosophila* posterior midgut and was found to be activated by VitD via a mechanism similar to that seen in mammals. The anti-aging effect of VitD has been well-established based on *in vitro* cell cultures and patient cases [60–63]. Recently, VitD has been prescribed for the treatment of colorectal cancer [64]. The results of the current study are consistent with previous studies on catalase-mutant flies, which showed that VitD reduced the effects of the age-dependent increase in ISC proliferation caused by high-level oxidative stress. Furthermore, for the first time, VitD was shown to reduce centrosome amplification, a hallmark of cancer. The evidence presented here demonstrates that VitD acts similarly in *Drosophila* and humans and exhibits anti-aging effects in the *Drosophila* intestine.

One limitation of this research is that the mechanisms of reduced cell proliferation, DNA damage, and centrosome amplification by VitD remain unknown. VitD and VDR act as transcription factors binding to VDRE at the antioxidant gene promoter region [37]. We conducted gene analyses to determine the VDRE binding sites in the *Drosophila* whole genome and identify the VitD target gene. Putative VDRE was located in the promoter region of several key genes. This finding will further contribute to the discovery of new anti-aging mechanisms by analyzing the anti-aging functions and roles of VitD.

In conclusion, this study provides direct evidence of the anti-aging role of the VitD/VDR pathway, involving protecting ECs during aging, and provides valuable insights for exploring the molecular mechanisms underlying enhanced healthy aging in *Drosophila*.

## MATERIALS AND METHODS

### Fly stock

All *Drosophila* fly stocks were reared at 25° C and provided with a standard cornmeal-molasses diet in a 12:12 light:dark (L:D) cycle. The standard meal consisted of 79.2% water, 10% sucrose, 7% cornmeal, 2% yeast, 1% agar, 0.5% propionic acid, and 0.3% bokinin. To avoid the overpopulation of larval in all vials, 50–60 adult flies per vial were transferred to new food vials every 2–3 days during their lifetime. *Oregon-R* represented wild-type flies. The model of intrinsic oxidative stress, *Catalase* heterozygous mutant flies (*Cat^n1^* mutant), was provided by the Bloomington Drosophila Stock Center (BDSC; Bloomington, IN, USA) [65]. The *Cat^n1^* mutant was chosen based on a previous study showing a gene dosage-dependent effect on the activity of catalase [65]. The Hr96 mutant was provided by the BDSC (#76592, #15856). The transgenic RNAi lines used were obtained from the Vienna Drosophila RNAi Center (VDRC; Vienna, Austria) and included *UAS-hr96-RNAi* (#330288) and *UAS-hr96-RNAi* (#10958). The *esg-Gal4,UAS-GFP/CyO* strain was provided by the Drosophila Genetic Resource Center (DGRC, Kyoto, Japan). The temperature-inducible ISC/EB-specific *esg-Gal4, tub-Gal80^ts^,UAS-GFP/CyO* (*esg^ts^*) was kindly provided by B. Ohlstein [5], while the temperature-inducible differentiated EC-specific *Myo1A-Gal80^ts^*, *Su-Gal80^ts^*, and *Pros-Gal80^ts^* flies were obtained from B.A. Edgar [53]. The *actin-GAL4/TM6B* was provided by the BDSC (#3954). The *esg^ts^>GFP* (*esg-Gal4*,*tub-Gal80^ts^,UAS-GFP+;+/+*) flies were obtained from a cross of *esg^ts^* females and *Oregon-R* males. *Myo^ts^>GFP* flies were obtained crossing *Myo1A-GAL4/CyO;UAS-GFP, tub-Gal80^ts^/TM6B* (*Myo^ts^*) females and *Oregon-R* males. *Su^ts^>GFP* flies were obtained from crossing *Oregon-R* males and *Su(H)-Gbe-GAL4,UAS-GFP/CyO; tub-Gal80^ts^/TM6B* (*Su^ts^*) females. *esg^ts^>GFP+hr96Ri* (#330288 or #10958) were obtained from crossing *UAS-hr96Ri* (#330288 or #10958) males and *esg^ts^* females. *Myo^ts^>GFP+hr96Ri* (#330288 or #10958) were obtained from crossing *UAS-hr96Ri* (#330288 or #10958) males and *Myo^ts^* females. *Su^ts^>GFP+hr96Ri* (#330288 or #10958) were obtained from crossing *UAS-hr96Ri* (#330288 or #10958) males and *Su^ts^* females. *pros^ts^>GFP+hr96Ri* (#330288 or #10958) were obtained from crossing *UAS-hr96Ri* (#330288 or #10958) males and *pros^ts^* females. The results described herein were those obtained using female flies.

### Temperature-controlled gene expression

The Gal80^ts^ technique was used for transgene expression at specific developmental stages and tissues [66]. Experimental flies were set and maintained at 22° C until adulthood. Following fly maintenance at 29° C for 4 or 7 days, their midguts were excised and dissected.

### Immunochemistry

For immunostaining using various antibodies, the entire intact adult gut was dissected and fixed at 25° C. Thereafter, the guts were fixed for 1 h in 4% formaldehyde (Sigma-Aldrich, St. Louis, MO, USA) for anti-GFP antibody staining. For co-immunostaining with primary antibody staining, the whole guts were fixed for 30 min in 4% paraformaldehyde in 1× phosphate-buffered saline (PBS) (Electron Microscopy Science, Hatfield, PA, USA). Subsequently, they were dehydrated for 5 min in 50, 75, 87.5, and 100% methanol, and rehydrated for 5 min in 50, 25, and 12.5% methanol in PBST (0.1% Triton X-100 in 1× PBS) for post-fixing. After washing thrice for 20 min with 1× PBST, the samples were incubated overnight with the primary antibodies at 4° C. After washing thrice for 20 min with 1× PBST, the samples were incubated at 25° C for 1 h with the secondary antibodies and 4′,6-diamidino-2-phenylindole (DAPI; 1;1000; Molecular Probes, Eugene, OR, USA), and washed again thrice for 20 min in 1× PBST. Subsequently, the samples were mounted using Vectashield (Vector Laboratories, Burlingame, CA, USA) and then analyzed using an Axioskop 2 Plus microscope (Carl Zeiss Inc., Göttingen, Germany). The number of PH3^+^ cells was counted in the whole midgut.

### Antisera

The following primary antibodies diluted in 1× PBST were used in this study: mouse anti-Dl, mouse anti-Pros, mouse anti-HP1 (Developmental Studies Hybridoma Bank, Iowa City, IA, USA), 1:200; mouse anti-GFP and rabbit anti-GFP (Molecular Probes,), 1:1000; rat anti-GFP (Nacalai Tesque Inc., Kyoto, Japan), 1:1000; rabbit anti-phospho-histone H3 (PH3; Millipore, Billerica, MA, USA), 1:1000; anti-Cleaved caspase-3 (Cell Signaling Technologies, Danvers, MA, USA), 1:1000; rabbit anti-γH2AvD (Rockland, Gilbertsville, PA, USA), 1:2000; mouse anti-γ-tubulin (Sigma-Aldrich), 1:1000; and anti-hVDR antibody (Thermo Fisher Scientific, Cleveland, OH, USA), 1:1000. The following secondary antibodies diluted in 1× PBST were used in this study: goat anti-rabbit FITC; goat anti-rabbit Cy3; goat anti-mouse FITC, goat anti-mouse Cy3, goat anti-rat FITC, and goat anti-rabbit Alexa Fluor® 647 (Jackson ImmunoResearch, West Grove, PA, USA), 1:400.

### VitD feeding assays

Three-day-old *Oregon-R*, *Myo^ts^>GFP* or *Myo^ts^>GFP+hr96Ri* flies were treated with 100 nM VitD (1α,25-Dihydroxyvitamin D_3_, D1530) (Sigma-Aldrich) [48] in standard feed media for 24 h or 7 days at 25 or 29° C, respectively.

### Quantitative analysis

For quantitative analysis of PH3^+^ cells, the cells were counted throughout the gut. To quantitatively analyze centrosome amplification, the number of γ-tubulin stained spots per PH3^+^ cell in the whole midgut was determined. For the quantitative analysis of Cleaved-Caspase 3^+^ cells, the number of cleaved Caspase 3^+^ cells per Myo^+^ cell in the posterior midgut was determined. The number of Dl^+^ cells per Myo^-^ small cell in the posterior midgut was determined for the quantitative analysis of Dl^+^ cells. To quantitatively analyze Pros^+^ cells, the number of Pros^+^ cells per total cell in the posterior midgut was determined. The quantified data are expressed as the mean ± standard error. Significant differences were determined using Student’s t-test. Sigma Plot 14.5 (Systat Software Inc., San Jose, CA, USA) was used to analyze standard error [16].

## Supplementary Material

Supplementary Figures

## References

[1] Rando TA. Stem cells, ageing and the quest for immortality. Nature. 2006; 441:1080–6. 10.1038/nature04958 16810243

[2] Spradling A, Drummond-Barbosa D, Kai T. Stem cells find their niche. Nature. 2001; 414:98–104. 10.1038/35102160 11689954

[3] Micchelli CA, Perrimon N. Evidence that stem cells reside in the adult Drosophila midgut epithelium. Nature. 2006; 439:475–9. 10.1038/nature04371 16340959

[4] Ohlstein B, Spradling A. The adult Drosophila posterior midgut is maintained by pluripotent stem cells. Nature. 2006; 439:470–4. 10.1038/nature04333 16340960

[5] Ohlstein B, Spradling A. Multipotent Drosophila intestinal stem cells specify daughter cell fates by differential notch signaling. Science. 2007; 315:988–92. 10.1126/science.1136606 17303754

[6] Sahai-Hernandez P, Castanieto A, Nystul TG. Drosophila models of epithelial stem cells and their niches. Wiley Interdiscip Rev Dev Biol. 2012; 1:447–57. 10.1002/wdev.36 23801493 PMC4924536

[7] Lee WC, Beebe K, Sudmeier L, Micchelli CA. Adenomatous polyposis coli regulates Drosophila intestinal stem cell proliferation. Development. 2009; 136:2255–64. 10.1242/dev.035196 19502486 PMC13471719

[8] Choi NH, Kim JG, Yang DJ, Kim YS, Yoo MA. Age-related changes in Drosophila midgut are associated with PVF2, a PDGF/VEGF-like growth factor. Aging Cell. 2008; 7:318–34. 10.1111/j.1474-9726.2008.00380.x 18284659 PMC2408640

[9] Biteau B, Hochmuth CE, Jasper H. JNK activity in somatic stem cells causes loss of tissue homeostasis in the aging Drosophila gut. Cell Stem Cell. 2008; 3:442–55. 10.1016/j.stem.2008.07.024 18940735 PMC3225008

[10] Park JS, Kim YS, Yoo MA. The role of p38b MAPK in age-related modulation of intestinal stem cell proliferation and differentiation in Drosophila. Aging (Albany NY). 2009; 1:637–51. 10.18632/aging.100054 20157545 PMC2806044

[11] Buchon N, Broderick NA, Poidevin M, Pradervand S, Lemaitre B. Drosophila intestinal response to bacterial infection: activation of host defense and stem cell proliferation. Cell Host Microbe. 2009; 5:200–11. 10.1016/j.chom.2009.01.003 19218090

[12] Buchon N, Broderick NA, Chakrabarti S, Lemaitre B. Invasive and indigenous microbiota impact intestinal stem cell activity through multiple pathways in Drosophila. Genes Dev. 2009; 23:2333–44. 10.1101/gad.1827009 19797770 PMC2758745

[13] Lee WJ. Bacterial-modulated host immunity and stem cell activation for gut homeostasis. Genes Dev. 2009; 23:2260–5. 10.1101/gad.1858709 19797765 PMC2758744

[14] Park JS, Lee SH, Na HJ, Pyo JH, Kim YS, Yoo MA. Age- and oxidative stress-induced DNA damage in Drosophila intestinal stem cells as marked by Gamma-H2AX. Exp Gerontol. 2012; 47:401–5. 10.1016/j.exger.2012.02.007 22387531

[15] Na HJ, Park JS, Pyo JH, Lee SH, Jeon HJ, Kim YS, Yoo MA. Mechanism of metformin: inhibition of DNA damage and proliferative activity in Drosophila midgut stem cell. Mech Ageing Dev. 2013; 134:381–90. 10.1016/j.mad.2013.07.003 23891756

[16] Park JS, Pyo JH, Na HJ, Jeon HJ, Kim YS, Arking R, Yoo MA. Increased centrosome amplification in aged stem cells of the Drosophila midgut. Biochem Biophys Res Commun. 2014; 450:961–5. 10.1016/j.bbrc.2014.06.085 24971546

[17] Beebe K, Lee WC, Micchelli CA. JAK/STAT signaling coordinates stem cell proliferation and multilineage differentiation in the Drosophila intestinal stem cell lineage. Dev Biol. 2010; 338:28–37. 10.1016/j.ydbio.2009.10.045 19896937

[18] Jiang H, Grenley MO, Bravo MJ, Blumhagen RZ, Edgar BA. EGFR/Ras/MAPK signaling mediates adult midgut epithelial homeostasis and regeneration in Drosophila. Cell Stem Cell. 2011; 8:84–95. 10.1016/j.stem.2010.11.026 21167805 PMC3021119

[19] Bond D, Foley E. Autocrine platelet-derived growth factor-vascular endothelial growth factor receptor-related (Pvr) pathway activity controls intestinal stem cell proliferation in the adult Drosophila midgut. J Biol Chem. 2012; 287:27359–70. 10.1074/jbc.M112.378018 22722927 PMC3431626

[20] Ren F, Wang B, Yue T, Yun EY, Ip YT, Jiang J. Hippo signaling regulates Drosophila intestine stem cell proliferation through multiple pathways. Proc Natl Acad Sci USA. 2010; 107:21064–9. 10.1073/pnas.1012759107 21078993 PMC3000252

[21] Shaw RL, Kohlmaier A, Polesello C, Veelken C, Edgar BA, Tapon N. The Hippo pathway regulates intestinal stem cell proliferation during Drosophila adult midgut regeneration. Development. 2010; 137:4147–58. 10.1242/dev.052506 21068063 PMC2990206

[22] Karpowicz P, Perez J, Perrimon N. The Hippo tumor suppressor pathway regulates intestinal stem cell regeneration. Development. 2010; 137:4135–45. 10.1242/dev.060483 21098564 PMC2990205

[23] Choi NH, Lucchetta E, Ohlstein B. Nonautonomous regulation of Drosophila midgut stem cell proliferation by the insulin-signaling pathway. Proc Natl Acad Sci USA. 2011; 108:18702–7. 10.1073/pnas.1109348108 22049341 PMC3219098

[24] Na HJ, Park JS, Pyo JH, Jeon HJ, Kim YS, Arking R, Yoo MA. Metformin inhibits age-related centrosome amplification in Drosophila midgut stem cells through AKT/TOR pathway. Mech Ageing Dev. 2015; 149:8–18. 10.1016/j.mad.2015.05.004 25988874

[25] Fan X, Liang Q, Lian T, Wu Q, Gaur U, Li D, Yang D, Mao X, Jin Z, Li Y, Yang M. Rapamycin preserves gut homeostasis during Drosophila aging. Oncotarget. 2015; 6:35274–83. 10.18632/oncotarget.5895 26431326 PMC4742104

[26] Zhou J, Florescu S, Boettcher AL, Luo L, Dutta D, Kerr G, Cai Y, Edgar BA, Boutros M. Dpp/Gbb signaling is required for normal intestinal regeneration during infection. Dev Biol. 2015; 399:189–203. 10.1016/j.ydbio.2014.12.017 25553980

[27] Osman D, Buchon N, Chakrabarti S, Huang YT, Su WC, Poidevin M, Tsai YC, Lemaitre B. Autocrine and paracrine unpaired signaling regulate intestinal stem cell maintenance and division. J Cell Sci. 2012; 125:5944–9. 10.1242/jcs.113100 23038775

[28] Li VS, Clevers H. Intestinal regeneration: YAP-tumor suppressor and oncoprotein? Curr Biol. 2013; 23:R110–2. 10.1016/j.cub.2012.12.021 23391384

[29] Lin G, Xu N, Xi R. Paracrine Wingless signalling controls self-renewal of Drosophila intestinal stem cells. Nature. 2008; 455:1119–23. 10.1038/nature07329 18806781

[30] Biteau B, Jasper H. EGF signaling regulates the proliferation of intestinal stem cells in Drosophila. Development. 2011; 138:1045–55. 10.1242/dev.056671 21307097 PMC3042864

[31] O’Brien LE, Soliman SS, Li X, Bilder D. Altered modes of stem cell division drive adaptive intestinal growth. Cell. 2011; 147:603–14. 10.1016/j.cell.2011.08.048 22036568 PMC3246009

[32] Li Z, Zhang Y, Han L, Shi L, Lin X. Trachea-derived dpp controls adult midgut homeostasis in Drosophila. Dev Cell. 2013; 24:133–43. 10.1016/j.devcel.2012.12.010 23369712

[33] Park JS, Na HJ, Pyo JH, Jeon HJ, Kim YS, Yoo MA. Requirement of ATR for maintenance of intestinal stem cells in aging Drosophila. Aging (Albany NY). 2015; 7:307–18. 10.18632/aging.100743 26000719 PMC4468312

[34] Randerson-Moor JA, Taylor JC, Elliott F, Chang YM, Beswick S, Kukalizch K, Affleck P, Leake S, Haynes S, Karpavicius B, Marsden J, Gerry E, Bale L, et al. Vitamin D receptor gene polymorphisms, serum 25-hydroxyvitamin D levels, and melanoma: UK case-control comparisons and a meta-analysis of published VDR data. Eur J Cancer. 2009; 45:3271–81. 10.1016/j.ejca.2009.06.011 19615888 PMC2786912

[35] Caini S, Boniol M, Tosti G, Magi S, Medri M, Stanganelli I, Palli D, Assedi M, Marmol VD, Gandini S. Vitamin D and melanoma and non-melanoma skin cancer risk and prognosis: a comprehensive review and meta-analysis. Eur J Cancer. 2014; 50:2649–58. 10.1016/j.ejca.2014.06.024 25087185

[36] Berridge MJ. Vitamin D cell signalling in health and disease. Biochem Biophys Res Commun. 2015; 460:53–71. 10.1016/j.bbrc.2015.01.008 25998734

[37] Warwick T, Schulz MH, Günther S, Gilsbach R, Neme A, Carlberg C, Brandes RP, Seuter S. A hierarchical regulatory network analysis of the vitamin D induced transcriptome reveals novel regulators and complete VDR dependency in monocytes. Sci Rep. 2021; 11:6518. 10.1038/s41598-021-86032-5 33753848 PMC7985518

[38] Fisk GJ, Thummel CS. Isolation, regulation, and DNA-binding properties of three Drosophila nuclear hormone receptor superfamily members. Proc Natl Acad Sci USA. 1995; 92:10604–8. 10.1073/pnas.92.23.10604 7479849 PMC40660

[39] Wilk R, Hu J, Krause HM. Spatial profiling of nuclear receptor transcription patterns over the course of Drosophila development. G3 (Bethesda). 2013; 3:1177–89. 10.1534/g3.113.006023 23665880 PMC3704245

[40] McKenna NJ, O’Malley BW. Combinatorial control of gene expression by nuclear receptors and coregulators. Cell. 2002; 108:465–74. 10.1016/s0092-8674(02)00641-4 11909518

[41] King-Jones K, Horner MA, Lam G, Thummel CS. The DHR96 nuclear receptor regulates xenobiotic responses in Drosophila. Cell Metab. 2006; 4:37–48. 10.1016/j.cmet.2006.06.006 16814731

[42] Horner MA, Pardee K, Liu S, King-Jones K, Lajoie G, Edwards A, Krause HM, Thummel CS. The Drosophila DHR96 nuclear receptor binds cholesterol and regulates cholesterol homeostasis. Genes Dev. 2009; 23:2711–6. 10.1101/gad.1833609 19952106 PMC2788327

[43] Sieber MH, Thummel CS. The DHR96 nuclear receptor controls triacylglycerol homeostasis in Drosophila. Cell Metab. 2009; 10:481–90. 10.1016/j.cmet.2009.10.010 19945405 PMC2803078

[44] Obniski R, Sieber M, Spradling AC. Dietary Lipids Modulate Notch Signaling and Influence Adult Intestinal Development and Metabolism in Drosophila. Dev Cell. 2018; 47:98–111.e5. 10.1016/j.devcel.2018.08.013 30220569 PMC6894183

[45] Jeon HJ, Kim YS, Park JS, Pyo JH, Na HJ, Kim IJ, Kim CM, Chung HY, Kim ND, Arking R, Yoo MA. Age-related change in γH2AX of Drosophila muscle: its significance as a marker for muscle damage and longevity. Biogerontology. 2015; 16:503–16. 10.1007/s10522-015-9573-0 25860864

[46] Amcheslavsky A, Jiang J, Ip YT. Tissue damage-induced intestinal stem cell division in Drosophila. Cell Stem Cell. 2009; 4:49–61. 10.1016/j.stem.2008.10.016 19128792 PMC2659574

[47] Park JS, Jeon HJ, Pyo JH, Kim YS, Yoo MA. Deficiency in DNA damage response of enterocytes accelerates intestinal stem cell aging in *Drosophila*. Aging (Albany NY). 2018; 10:322–38. 10.18632/aging.101390 29514136 PMC5892683

[48] Halicka HD, Zhao H, Li J, Traganos F, Studzinski GP, Darzynkiewicz Z. Attenuation of constitutive DNA damage signaling by 1,25-dihydroxyvitamin D3. Aging (Albany NY). 2012; 4:270–8. 10.18632/aging.100450 22498490 PMC3371762

[49] Erez N, Israitel L, Bitman-Lotan E, Wong WH, Raz G, Cornelio-Parra DV, Danial S, Flint Brodsly N, Belova E, Maksimenko O, Georgiev P, Druley T, Mohan RD, Orian A. A Non-stop identity complex (NIC) supervises enterocyte identity and protects from premature aging. Elife. 2021; 10:e62312. 10.7554/eLife.62312 33629655 PMC7936876

[50] Lu YX, Regan JC, Eßer J, Drews LF, Weinseis T, Stinn J, Hahn O, Miller RA, Grönke S, Partridge L. A TORC1-histone axis regulates chromatin organisation and non-canonical induction of autophagy to ameliorate ageing. Elife. 2021; 10:e62233. 10.7554/eLife.62233 33988501 PMC8186904

[51] Nan Y, Lin J, Cui Y, Yao J, Yang Y, Li Q. Protective role of vitamin B6 against mitochondria damage in Drosophila models of SCA3. Neurochem Int. 2021; 144:104979. 10.1016/j.neuint.2021.104979 33535071

[52] Khayami R, Goltzman D, Rabbani SA, Kerachian MA. Epigenomic effects of vitamin D in colorectal cancer. Epigenomics. 2022; 14:1213–28. 10.2217/epi-2022-0288 36325830

[53] Jiang H, Patel PH, Kohlmaier A, Grenley MO, McEwen DG, Edgar BA. Cytokine/Jak/Stat signaling mediates regeneration and homeostasis in the Drosophila midgut. Cell. 2009; 137:1343–55. 10.1016/j.cell.2009.05.014 19563763 PMC2753793

[54] Biteau B, Jasper H. Slit/Robo signaling regulates cell fate decisions in the intestinal stem cell lineage of Drosophila. Cell Rep. 2014; 7:1867–75. 10.1016/j.celrep.2014.05.024 24931602 PMC4086754

[55] Lind A, Wängberg B, Ellegård L. Vitamin D and vitamin B12 deficiencies are common in patients with midgut carcinoid (SI-NET). Eur J Clin Nutr. 2016; 70:990–4. 10.1038/ejcn.2016.40 27026421

[56] Fagan R, Bokhari SSN, Inayat F. Vitamin D and vitamin B_12_ deficiencies in patients with small intestinal carcinoid tumour: is opioid use disorder a confounding factor in the diagnosis? BMJ Case Rep. 2019; 12:e227430. 10.1136/bcr-2018-227430 30878964 PMC6424257

[57] Altieri B, Barrea L, Modica R, Bottiglieri F, de Cicco F, Muscogiuri G, Circelli L, Savarese G, Di Somma C, Savastano S, Colao A, Faggiano A. Vitamin D deficiency and tumor aggressiveness in gastroenteropancreatic neuroendocrine tumors. Endocrine. 2022; 75:623–34. 10.1007/s12020-021-02869-w 34533768

[58] Fang Y, Song H, Huang J, Zhou J, Ding X. The clinical significance of vitamin D levels and vitamin D receptor mRNA expression in colorectal neoplasms. J Clin Lab Anal. 2021; 35:e23988. 10.1002/jcla.23988 34651346 PMC8605155

[59] Park JS, Kim YJ. Anti-Aging Effect of the Ketone Metabolite β-Hydroxybutyrate in *Drosophila* Intestinal Stem Cells. Int J Mol Sci. 2020; 21:3497. 10.3390/ijms21103497 32429095 PMC7278929

[60] Mitsuo T, Nakao M. [Vitamin D and anti-aging medicine]. Clin Calcium. 2008; 18:980–5. 18591751

[61] Li W, Che X, Chen X, Zhou M, Luo X, Liu T. Study of calcitriol anti-aging effects on human natural killer cells *in vitro*. Bioengineered. 2021; 12:6844–54. 10.1080/21655979.2021.1972076 34546851 PMC8806577

[62] Sosa-Díaz E, Hernández-Cruz EY, Pedraza-Chaverri J. The role of vitamin D on redox regulation and cellular senescence. Free Radic Biol Med. 2022; 193:253–73. 10.1016/j.freeradbiomed.2022.10.003 36270517

[63] Giustina A, Bouillon R, Dawson-Hughes B, Ebeling PR, Lazaretti-Castro M, Lips P, Marcocci C, Bilezikian JP. Vitamin D in the older population: a consensus statement. Endocrine. 2023; 79:31–44. 10.1007/s12020-022-03208-3 36287374 PMC9607753

[64] Hamada T, Liu L, Nowak JA, Mima K, Cao Y, Ng K, Twombly TS, Song M, Jung S, Dou R, Masugi Y, Kosumi K, Shi Y, et al. Vitamin D status after colorectal cancer diagnosis and patient survival according to immune response to tumour. Eur J Cancer. 2018; 103:98–107. 10.1016/j.ejca.2018.07.130 30219720 PMC6195453

[65] Griswold CM, Matthews AL, Bewley KE, Mahaffey JW. Molecular characterization and rescue of acatalasemic mutants of Drosophila melanogaster. Genetics. 1993; 134:781–8. 10.1093/genetics/134.3.781 8349109 PMC1205515

[66] McGuire SE, Roman G, Davis RL. Gene expression systems in Drosophila: a synthesis of time and space. Trends Genet. 2004; 20:384–91. 10.1016/j.tig.2004.06.012 15262411

