# Supplementary material for: The anti-aging effect of vitamin D and vitamin D receptor in Drosophila midgut
Source: Aging (Albany NY). 2024 Feb 7;16(3):2005–25. doi: 10.18632/aging.205518 (PMC10911382; doi:10.18632/aging.205518)
Supplement: Supplementary Figures [file aging-16-205518-s001.pdf]

SUPPLEMENTARY FIGURES

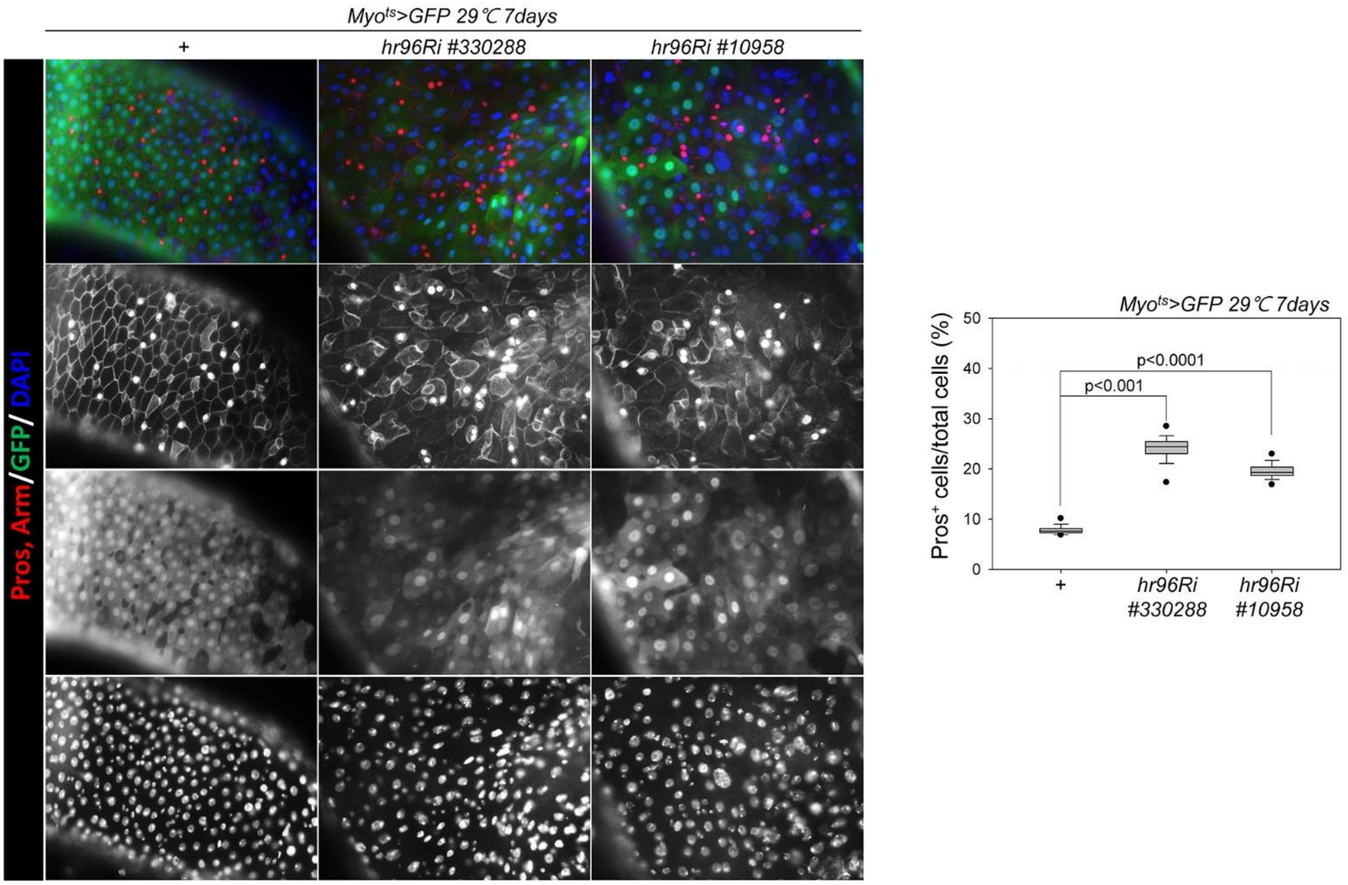

Supplementary Figure 1. EC-specific VDR knockdown increases the number of Pros^{+} cells. Flies carrying the *Myo^{ts}>GFP*, *Myo^{ts}>GFP+VDRRi* #330288, *Myo^{ts}>GFP+VDRRi* #10958 genotypes were cultured at 29° C for 7 days. The guts of flies were dissected and labeled with anti-GFP (green), anti-Pros (red), and anti-Arm (red) antibodies and DAPI (blue). Original magnification is 200×. Frequency of Pros^{+} cell per total cells. Three-day-old females were shifted to 29° C for 7 days and dissected guts were immunostained with anti-GFP (green), anti-Pros (red), and anti-Arm (red) antibodies and DAPI (blue). The Pros^{+} cell numbers were counted in the total cells of these guts. Data (mean ± standard error) in the *Myo^{ts}>GFP*, *Myo^{ts}>GFP+VDRRi* #330288, *Myo^{ts}>GFP+VDRRi* #10958 flies were collated from 5533, 3645, and 4075 total cells of 19, 17, and 18 guts, respectively. *P*-values were calculated using the Student's *t*-test. $P < 0.0001$ and $p < 0.001$ compared to that of the *Myo^{ts}>GFP* flies.

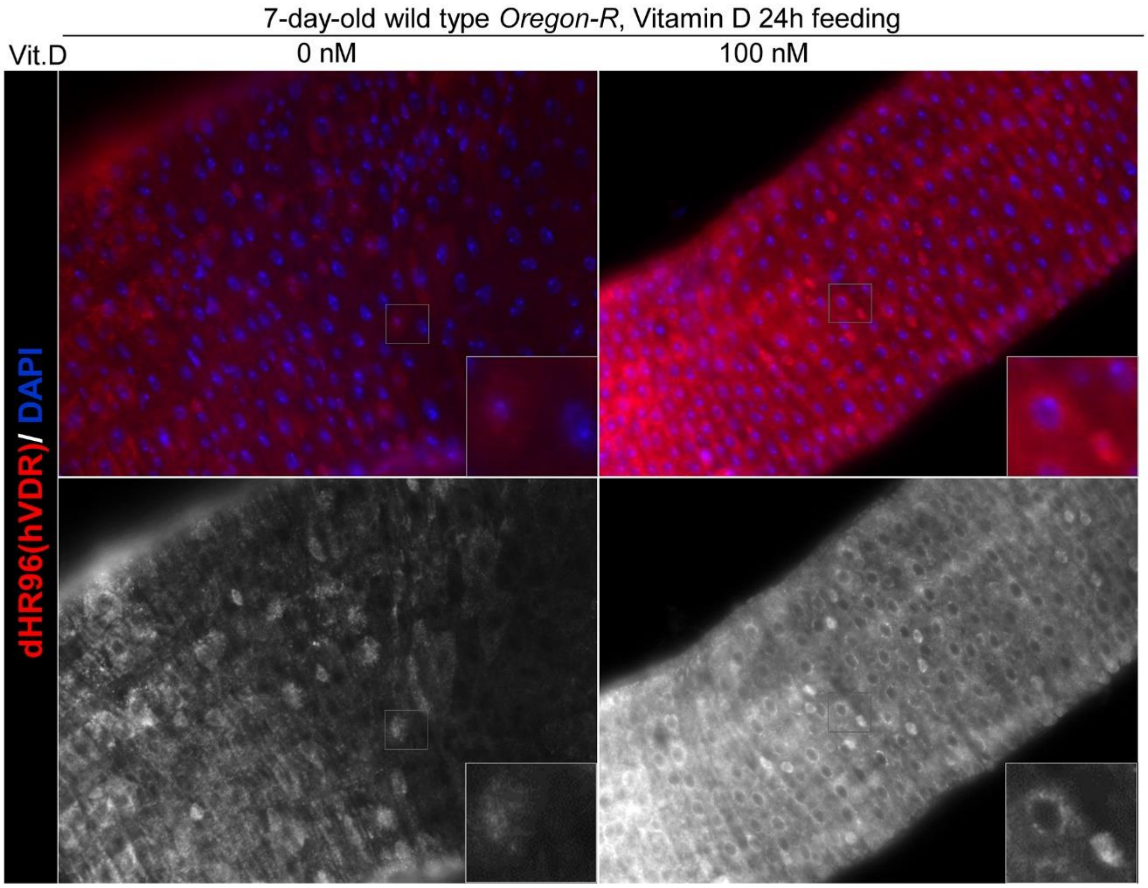

Supplementary Figure 2. Effect of VitD on VDR localization in *Drosophila* intestine. Gut from 3-day-old wild-type female flies without (0 nM) or with (100 nM) active VitD (1α,25-Dihydroxyvitamin D_3) feeding for seven 24 h, were stained with anti-hVDR antibody (red) and DAPI (blue). Original magnification is 200×. Gray squares in panels indicate magnified regions below.

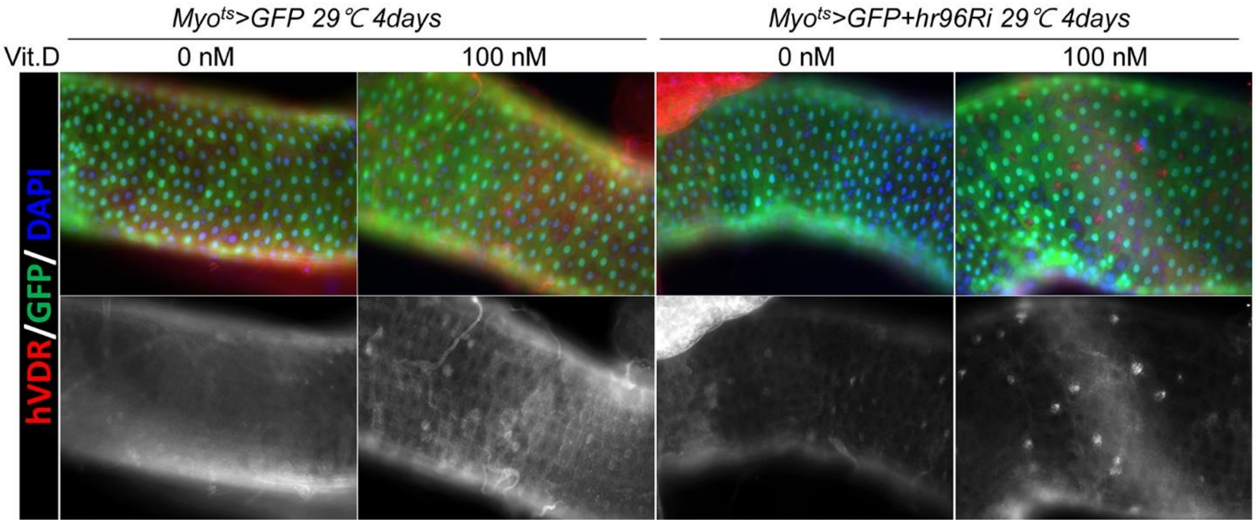

Supplementary Figure 3. Hr96 acts as VDR during VitD feeding in *Drosophila* intestine. Guts from 3-day-old *Myo^{ts}>GFP and Myo^{ts}>GFP+VDRRi* flies, without or with 100 nM VitD feeding at 29° C for 4 days, were stained with anti-hVDR (red), anti-GFP (green), and DAPI (blue). Original magnification is 400×.

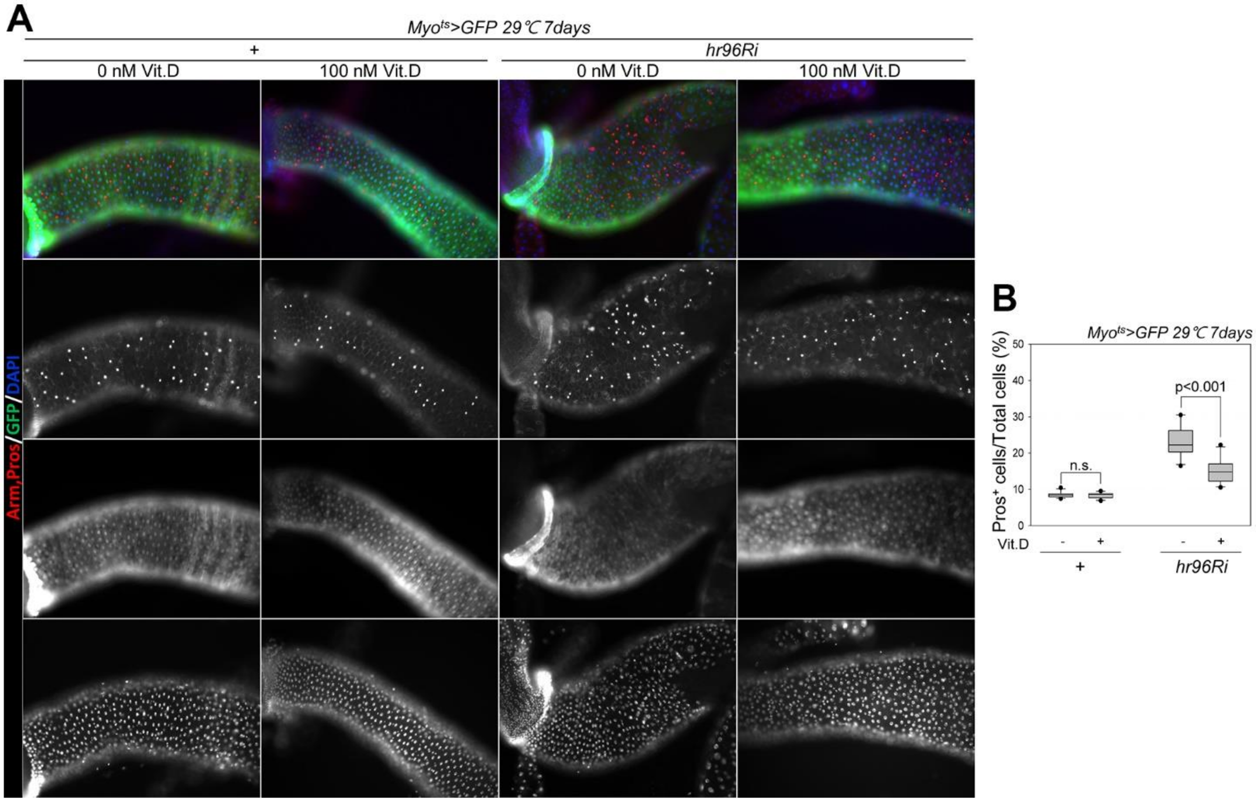

Supplementary Figure 4. EC-specific VDR knockdown-induced increase of EE cell number reduced by VitD. (**A**) Guts from 3-day-old *Myo^{ts}>GFP and Myo^{ts}>GFP+VDRRi* flies, without or with 100 nM VitD feeding at 29° C for 7 days, were stained with anti-Pros (red), anti-Arm (red), anti-GFP (green), and DAPI (blue). Original magnification is 400×. (**B**) Frequency of Pros$^+$ cells per total cells. Three-day-old females were shifted to 29° C for 7 days, and dissected guts were immunostained with anti-Pros (red), anti-Arm (red), anti-GFP (green), and DAPI (blue). The Pros$^+$ cell numbers were recorded with respect to the total cells of these guts. Data (mean ± standard error) in 10-day-old *Myo^{ts}>GFP* and *Myo^{ts}>GFP+VDRRi* flies without VitD feeding were collated from 4688 and 5050 total cells of 12 and 13 guts, respectively. Data (mean ± standard error) in 10-day-old *Myo^{ts}>GFP* and *Myo^{ts}>GFP+VDRRi* flies with VitD feeding were collated from 5036 and 4768 mitotic cells of 12 and 12 guts, respectively. n.s., indicates not significant (p>0.05). P-values were calculated using the Student's t-test. P < 0.001 compared to that of the *Myo^{ts}>GFP+VDRRi* flies without VitD.

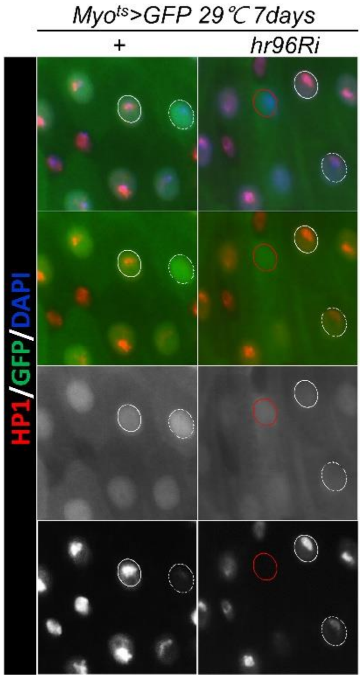

Supplementary Figure 5. EC-specific VDR knockdown causes the loss of heterochromatin marker (HP1) in *Drosophila* intestinal enterocytes. Flies carrying the *Myo^{ts}>GFP* and *Myo^{ts}>GFP+VDRRi* genotypes were cultured at 29° C for 7 days. The guts of flies were dissected and labeled with anti-GFP (green) and anti-HP1 (red) antibodies and DAPI (blue). Circles indicate nuclei of ECs (GFP^{+} cells). White circle, condensed pattern of HP1; White dashed circle, dispersed and weak pattern of HP1; Red circle, loss of HP1. Original magnification is 200×.

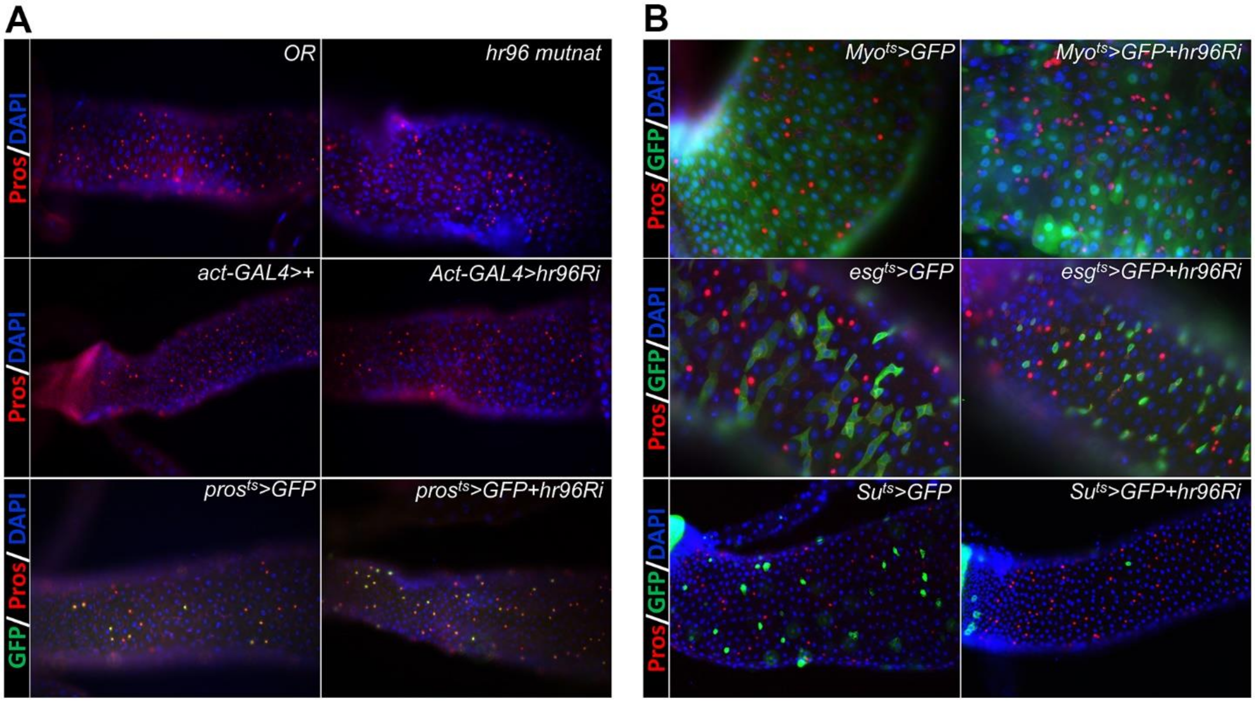

Supplementary Figure 6. Intestinal cell type-specific differential effect of VDR on the number of Pros^{+} cells. (**A**) Wile-type, *hr96* mutant female, and flies carrying the *act-GAL4>+*, *act-GAL4>VDRRi*, *pros^{ts}>GFP*, and *pros^{ts}>GFP+VDRRi* genotypes were cultured at 29° C for 7 days. (**B**) Flies carrying the *Myo^{ts}>GFP*, *Myo^{ts}>GFP+VDRRi*, *esg^{ts}>GFP*, *esg^{ts}>GFP+VDRRi*, *Su^{ts}>GFP*, or *Su^{ts}>GFP+VDRRi* genotypes were cultured at 29° C for 7 days. The guts of flies were dissected and labeled with anti-GFP (green), and anti-Pros (red), and anti-Arm (red) antibodies and DAPI (blue). Original magnification is 400×.

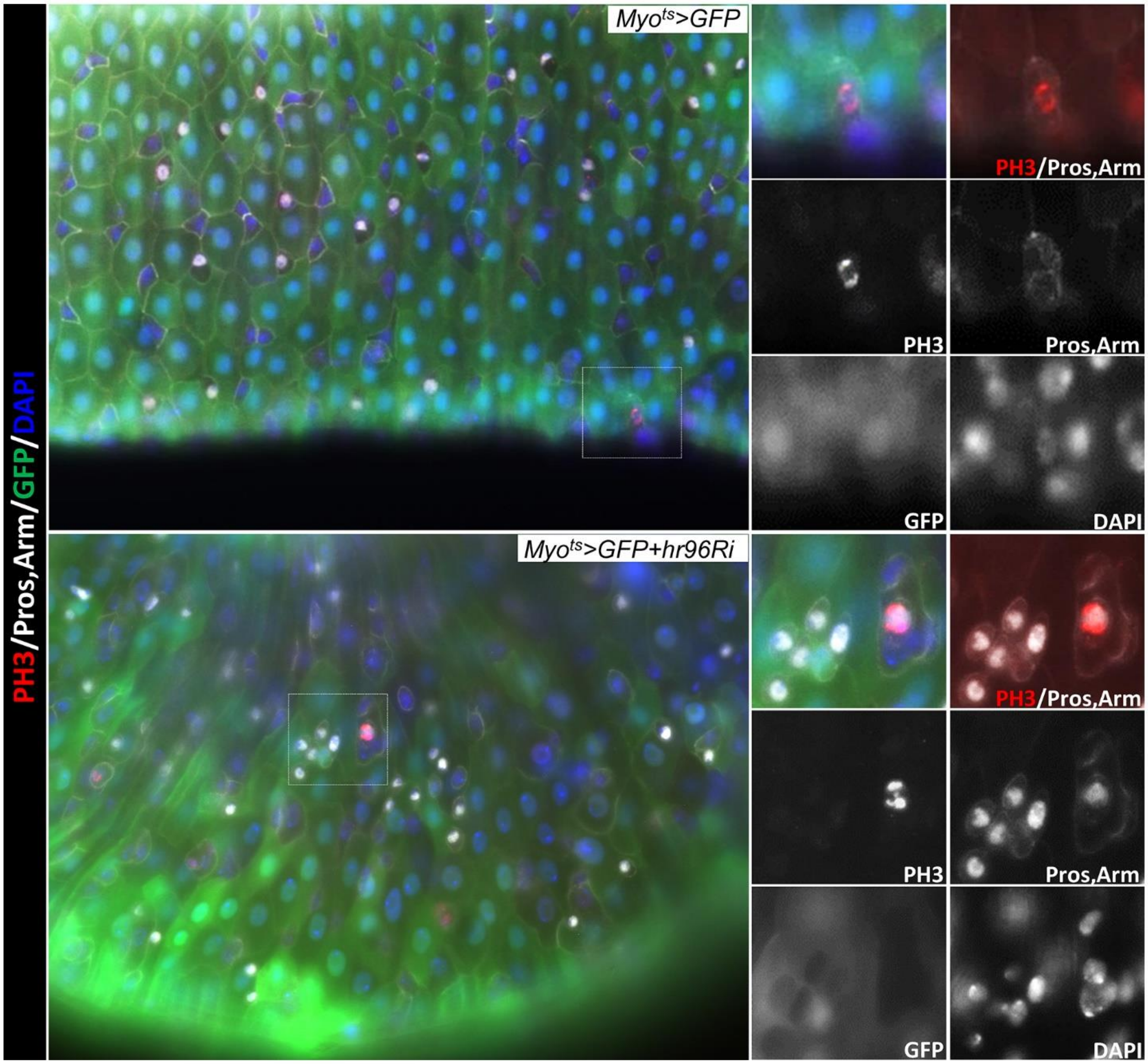

Supplementary Figure 7. EC-specific knockdown of VDR results in dividing EE cell formation. Flies carrying the *Myo^{ts}>GFP* and *Myo^{ts}>GFP+VDRRi* genotypes were cultured at 29° C for 7 days. The guts of flies were dissected and labeled with anti-GFP (green), anti-Pros (white), anti-Arm (white), and anti-PH3 (red) antibodies and DAPI (blue). Gray squares in panels indicate magnified regions. Original magnification is 200×.
